# Papillary Vessel Density Changes after Intravitreal Anti-VEGF Injections in Hypertensive Patients with Central Retinal Vein Occlusion: An Angio-OCT Study

**DOI:** 10.3390/jcm8101636

**Published:** 2019-10-06

**Authors:** Michele Nicolai, Alessandro Franceschi, Serena De Turris, Alessandro Rosati, Vittorio Pirani, Cesare Mariotti

**Affiliations:** Eye Clinic, Polytechnic University of Marche, via Conca 61, 60126 Ancona, Italy; michele.nicolai@hotmail.it (M.N.); serena_deturris@hotmail.it (S.D.T.); alessandro.rosati8@gmail.com (A.R.); vittorio.Pirani@ospedaliriuniti.marche.it (V.P.); cesare.mariotti@ospedaliriuniti.marche.it (C.M.)

**Keywords:** central retinal vein occlusions, anti-VEGF, Optical coherence tomography angiography, Peripapillary vessel density

## Abstract

Purpose: To investigate papillary microvascular changes in patients affected by macular edema due to Central Retinal Vein Occlusions (CRVO) after anti-Vascular Endothelial Growth Factor (VEGF) therapy. Methods: Prospective analysis of papillary and peripapillary vessel density (VD) changes in 18 eyes of 18 hypertensive patients affected by CRVO before and after the loading-phase of intravitreal Ranibizumab (IVR) injections. Data were quantitatively measured by optical coherence tomography (OCT) and optical coherence tomography angiography (OCTA) before as well as 1 month and 4 months after injections. The correlation between post-treatment best-corrected visual acuity (BCVA) and changes in the retinal microvasculature evaluated by OCTA was assessed. Results: 18 eyes of 18 consecutive patients with a known history of arterial hypertension and affected by an acute CRVO episode were enrolled. Central macular thickness (CMT) was significantly reduced after IVR injections (*p* < 0.001), while mean BCVA improved from 0.70 ± 0.26 logarithm of the minimal angle of resolution (logMAR) units at baseline to 0.25 ± 0.18 logMAR units after 4 months (*p* < 0.001). VD inside disc and peripapillary significantly increased (*p* < 0.001 and *p* = 0.01, respectively) after treatment. Conclusions: OCTA showed VD increase in the papillary area in patients affected by CRVO after anti-VEGF therapy. This area could represent a new region of interest to study microvasculature changes concomitant with severe macular edema.

## 1. Introduction

Depending on the location of the occlusion the Retinal Vein Occlusion (RVO) can be classified as being branch, central, or hemiretinal; Branch Retinal Vein Occlusions (BRVOs) are more common than Central Retinal Vein Occlusions (CRVOs) [1,2]. RVO can be caused by different systemic pathologies like diabetes mellitus, hypertension, cardiovascular diseases, increased body mass index (BMI), reduced High-Density Lipoprotein (HDL) levels, thyroid disorders, peptic ulcer, and smoking. In addition, Kaderli et al. [3] found out that arterial stiffness as measured by pulse wave velocity and aortic distensibility was abnormal in BRVO patients, in comparison with both healthy and hypertensive controls. Hypertension and atherosclerosis are the major risk factors for CRVO and play significant roles in its pathophysiology [4]. They lead to hardening and thickening of the arteriolar wall, and the thickened artery compresses the vein within a common adventitial sheath inducing turbulence, endothelial damage, and thrombosis of the retinal venous tree [5,6]. There are also ocular risk factors such as glaucoma, shorter axial length, focal arteriolar narrowing, and arteriovenous (AV) nicking [7,8] The diagnosis of acute RVO is performed by fundoscopy, the characteristic signs are flame hemorrhages, dot and blot hemorrhages, cotton wool spots, hard exudates, retinal edema, and dilated tortuous veins [7]. Macular edema and retinal non perfusion are usually the most threatening complications in RVOs [9]. The gold standard for quantitatively and qualitatively assessing structural macular changes is spectrum-domain optical coherence tomography (SD-OCT). Perfused vascular retinal network is better studied with fluorescein angiography (FA), which is able to detect retinal ischemia and neovascularization [1,10]. Optical coherence tomography angiography (OCTA) is a new technique that allows us to study retinal microvasculature by calculating the difference between a static tissue (vessel) and a dynamic one (red blood cells) in a non-invasive way [11]. One of the most common types of OCTA imaging acquisition systems is based on a split spectrum amplitude decorrelation algorithm (SSADA) [12]. The movement of red blood cells within the retinal capillaries is used as an intrinsic contrast medium to generate flow imaging, independently of the direction of movement [13]. At the same time, all static (structural) information is removed by the software. Thus, in contrast to fluorescein angiography (FA), data in OCTA are derived from the red blood cells rather than from plasma [14]. OCTA displays the eye microvasculature and its 3-dimensional structure in great detail and high resolution without dye injection. This allows a detailed visualization of the superficial and deep plexus, making it possible to evaluate vessel density and vasculature in different retinal layers [15].

Current standard of care for the treatment of patients with macular edema induced by RVOs is intravitreal anti-VEGF therapy or dexamethasone implant [1]. So far, the study of vascular density in patients affected by RVO, measured with OCTA, has been limited mainly to the analysis of the fovea and FAZ. Several studies have documented that FAZ enlargement is correlated with visual acuity impairment [16] and that microcirculation alterations relate to the development of macular edema in patients with RVO [17]. Studies performed with OCTA have shown that both superficial and deep parafoveal Vessel Density (VD) can be decreased in RVO [18,19]. Anti-VEGF therapy reduces the size of the retinal non-perfused area (NPA) and improves retinal blood flow, especially in the deep capillary layer [20,21]. On the other hand, many previous studies assessed that VD remained statistically unchanged after anti-VEGF therapy [22,23,24].

Recent studies have shown that the vascular perfusion status could affect visual recovery after the resolution of macular edema. Visual improvement after the treatment of macular edema significantly correlates with better retinal perfusion and lower rates of retinal ischemia in patients with RVO.

Peripapillary vessel density is a parameter whose role has been deeply investigated in glaucoma and recently in Diabetic Retinopathy (DR) [25]. Many studies observed a quantitative vessel density reduction after glaucomatous damage was noticed [22,23]. Other anatomical changes that occur in glaucoma are: retinal ganglion cells axons injury; thinning of the retinal nerve fiber layer (RNFL) thickness; intrapapillary fibers loss (“cupping”); posterior displacement of the lamina cribrosa. These complex modifications probably lead to circulatory changes, resulting in an ischemic insult [26,27].

At present, there are no studies about peripapillary vessel network changes in eyes affected by CRVO. The purpose of this study was to evaluate peripapillary vessel density changes in patients with CRVO before and after intravitreal anti-VEGF injections using OCTA technology.

## 2. Experimental Section

This was a prospective, board-approved single-center observational clinical study (approval code) which enrolled consecutively 18 patients (7 women, 11 men) who underwent an acute CRVO episode between September 2018 and April 2019. The procedures of this study were in accordance with the Declaration of Helsinki. Written consent was obtained from all individual participants included in this paper, and it was approved by the local Institutional Review Board (IRB). All patients were examined at our Department and were treated with a loading phase of 3 intravitreal injections of Ranibizumab (IVR; Lucentis, Genentech, San Francisco, CA, USA) with monthly interval. Inclusion criteria were: (1) a recent episode of CRVO (less than 15 days), (2) known history of arterial hypertension, (3) presence of macular edema, (4) a minimum follow up of 4 months. Exclusion criteria were: (1) macular edema due to causes other than RVO, (2) poor quality OCTA images, (3) lack of follow up control exams for low patient compliance, (4) glaucoma. None of the patients presented other ocular diseases. All the 18 patients were treatment-naive. A detailed antihypertensive drug history and compliance was noted.

All patients sustained a complete ophthalmic examination in both eyes including measurement of Best-Correct Visual Acuity (BCVA), Goldman tonometry, slit-lamp biomicroscopy, and indirect fundus ophthalmoscopy, OCT, RNFL thickness, OCT Angiography (Optovue RTVue XR Avanti; Optovue, Inc., Fremont, CA, USA) and Fluorangiography (Heidelberg Retina Angiograph 2 (HRA2); Heidelberg Engineering, Heidelberg, Germany). The BCVA decimal visual acuities were converted to the logarithm of the minimal angle of resolution (logMAR) units for the statistical analyses. All the ophthalmic examination and image acquisition were performed at the same hour to reduce circadian IOP (Intra-Ocular-Pressure) variation and blood-flow changes. Adherence to anti-hypertensive therapy was checked before proceeding to data acquisition.

### 2.1. Imaging Protocol

The AngioVue system (Optovue RTVue XR Avanti; Optovue, Inc., Fremont, CA, USA) was used for all OCT and OCTA images before and after anti-VEGF therapy. Central macular thickness (CMT) was automatically measured in the macular map centered to the fovea. Mean RNFL thickness parameters were obtained from automatic software measurements.

**Figure 1:**
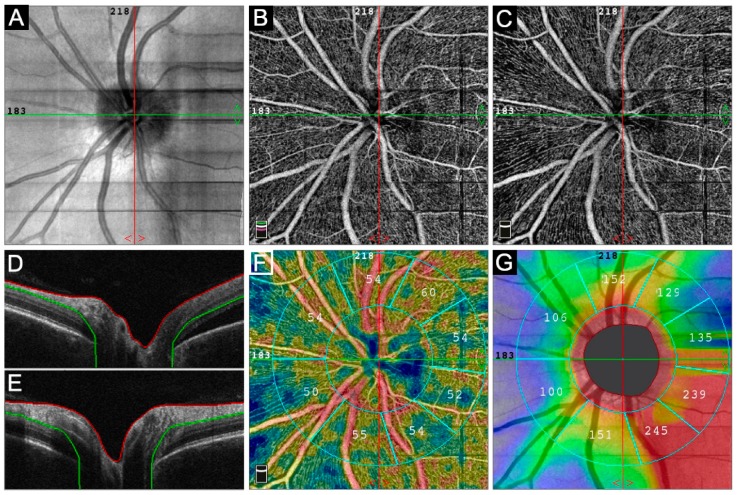
The optic nerve parameters acquired. Infrared image of the disc area (**A**). Optical Coherence Tomography (OCT) and Optical Coherence Tomography-Angiography (OCT-A) scans above outer plexiform layer (**B**,**D**) and between inner limiting membrane and nerve fiber layer (**C**,**E**). Inside disc Vessel Density (VD) (**F**), measured inside the optic disc boundary and peripapillary VD, calculated for the region of 750 μm-wide elliptical annulus extending from the optic disc boundary. Nerve fiber thickness map, including Retinal Nerve Fiber Layer (RNFL) (**G**).

We measured the papillary retinal vessel density, which could be defined as the total length of perfused vasculature per unit area in a region of measurement. It was automatically analyzed in the Radical Peripapillary Capillary (RPC) segment (4.5 × 4.5-mm) and divided in three segments: whole image, inside disc, and peripapillary. Inside disc vessel density was measured for the region inside the optic disc boundary (Figure 1). Whole en face vessel density was measured for the entire 4.5 × 4.5 mm image, while whole peripapillary vessel density was calculated for the region of 750 µm-wide elliptical annulus extending from the optic disc boundary. We excluded poor-quality images from the final OCTA analysis with the following characteristics: (1) signal strength less than 48; (2) motion artifacts visible as irregular vessel pattern or disc boundaries on OCTA scans; (3) poor clarity due to the presence of cataracts, corneal opacities, vitreous alterations, or severe papillary edema.

### 2.2. Statistical Analysis

Statistical evaluation was performed using Statistical Package for Social Sciences (version 17.0, SPSS Inc., Armonk, NY, USA). All results of quantitative variables, including age, BCVA, CMT, RNFL and VD, were expressed as the mean ± standard deviations. Gender (male) was expressed as a percentage.

Mean and standard deviation values were calculated for continuous variables after checking for normal distribution of the data, while frequency and percentage were calculated for categorical variables. A t-test was used for continuous variables.

Demographic characteristics and clinical data are summarized in Table 1.

## 3. Results

18 eyes of 18 consecutive hypertensive patients with treatment-naive macular edema secondary to CRVO before and after a loading phase of 3 ranibizumab injections were analyzed. The mean patient age was 56.09 ± 16.00; the group included 11 males and 7 females. Mean logMAR BCVA was 0.70 ± 0.26 at baseline, 0.34 ± 0.17 one month after the first injection and 0.25 ± 0.18 after four months. Differences in BCVA between baseline and 1-month post-operative and between baseline and 4 months post-operative were statistically significative with a *p*-value of less than 0.001 in both cases.

VD values (%) at baseline, one month and four months after treatment were, respectively: 44.64 ± 2.70, 45.34 ± 2.63 and 45.75 ± 4.56 in the whole image analysis; 45.16 ± 2.33, 45.71 ± 2.03 and 47.08 ± 2.58 for the inside disc; 43.84 ± 1.74, 44.78 ± 2.11 and 45.58 ± 2.86 for the peripapillary. Inside disc VD differences between baseline and four-months post-operative group were statistically significant with a *p*-value < 0.001. Peripapillary VD difference between the baseline and 1-month group and baseline and 4-months group were both statistically significant with a *p*-value of 0.04 and 0.01, respectively.

**Figure 2:**
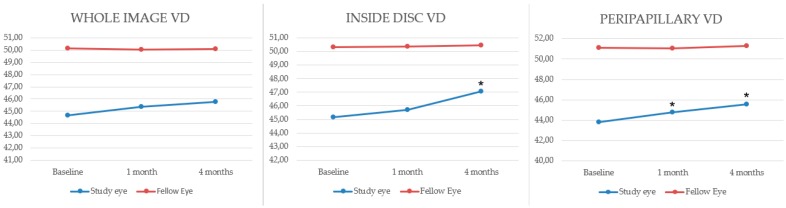
Comparison between the study eye (blue) and the fellow eye (red). Whole image, inside disc and peripapillary VD differences (percentages) were highly statistically significant between the affected eye and the fellow eye at baseline, 1- and 4-months post-treatment (*p* < 0.001). In the affected eyes, statistically significant parameters changes (*p* < 0.05) are shown with an asterisk. Abbreviations: CRVO = central retinal vein occlusion; VD = vessel density.

In the fellow eyes, differences between VD values at baseline and in the post-treatment period were not statistically significant (Table 1). Whole image, inside disc and peripapillary VD differences were highly statistically significant between the affected eye and the fellow eye at baseline, 1 and 4 months post-treatment (*p* < 0.001), data are shown in Figure 2.

**Figure 3:**
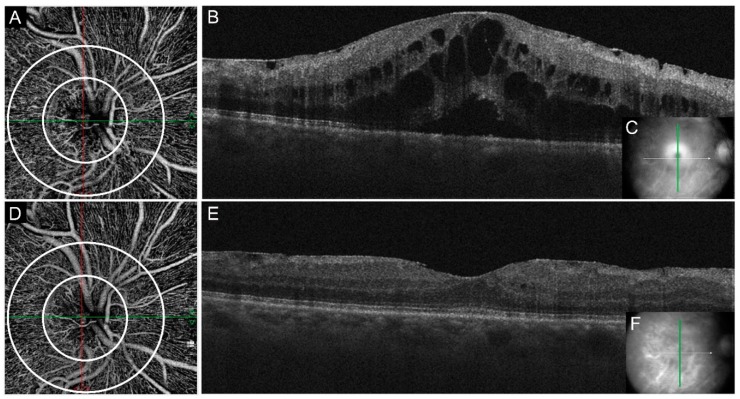
Optic nerve vessel density changes before (**A**) and after 4-months (**D**) from intravitreal anti-Vascular Endothelial Growth Factor (VEGF) injection. Inside disc and peripapillary VD are highlighted by white circles. Follow-up OCT vertical scans show a reduction of macular edema from baseline (**B**,**C**) to 4-months (**E**,**F**).

Central macular thickness (µm) was 772.61 ± 199.54 pre-treatment, 435.56 ± 105.42 after one month and 421.28 ± 111.66 after four months (*p* < 0.001) (Figure 3). On fluorescein angiography, 2 of 18 patients presented a non-perfused area larger than 10-disc areas. RNFL thickness analysis did not show any statistical significance before and after the treatment (*p* = 0.3; *p* = 0.05).

## 4. Discussion

OCTA features in RVO have been widely shown by several authors [28,29]. OCTA represents a better imaging modality to detect retinal cystoid spaces and perifoveal capillary arcade in comparison with FA. FAZ enlargement and capillary dropouts after RVO damage have been demonstrated. Coscas et al. [29] have shown a more frequent reduction of VD in the deep capillary plexus (DCP) compared with the superficial capillary plexus (SCP) after RVO; it seemed to be correlated with a degree of peripheral ischemia, evaluated by means of FA [30]. Studies of animal model of RVO showed that the SCP has a greater perfusion than DCP because it is directly connected to the retinal arterioles. On the other hand, DCP is primarily formed by venous collecting channels and could be more susceptible to venous occlusion [20,30]. OCTA can be useful to evaluate microvascular changes before an occlusive event, Adhi et al. showed how the eyes of RVO patients present diminished vascular perfusion of DCP compared with healthy controls [31].

The influence of anti-VEGF therapy on retinal perfusion has not a clear interpretation, various studies demonstrated conflicting results [20,32,33]. Suzuki et al. [20] reported improved perfusion areas in DCP and SCP after anti-VEGF therapy in patients with RVO. In contrast, other authors [32,33] have shown that there is no reperfusion after anti-VEGF but instead that the capillaries already present became visible in eyes after diabetic macular edema (DME) resolution.

Recent studies [34,35] focused on vessel density changes and macular perfusion in RVO after anti-VEGF using OCTA technology. Andrew Winegarner et al. [34] found no significant changes in mean VD 12 months after anti-VEGF injections in patients with RVO, although higher VD and a smaller FAZ were associated with better BCVA. Other authors [24,35,36] confirmed no significant variation of macular VD after treatment.

A reason for these inconsistent anatomical findings compared with significant functional benefit regarding VD in RVO is not completely understood; but some considerations have been made. A consistent limitation of these studies is the image quality acquisition when macular edema is present. Severe macular edema has some degrees of interference with accurate interpretations of OCTA parameters, due to probable signal attenuation and difficulty in precise retinal plexus distinction of the automated segmentation tool. Furthermore, microvascular macular changes could distort OCTA quantitative results. On the one hand, when a greater amount of macular edema is detected, vessel density could be overestimated due to vessel dilatation. On the other hand, if the blood flow of the dilatated vessel is below the detection limit of 0.3 mm/second on OCTA [37], it is read as a cystoid space instead of a vessel venous engorgement.

We mentioned above that it is possible to hypothesize that masking effect or retinal vessel displacement due to edema could justify the reappearance of the vessels that were poorly identified before treatment [32,33,34,35].

As the macular region could be a burdensome site to investigate when severe macular edema is present, other spots should be considered. Some authors [13,35,38] examined the choroidal layer and demonstrated that the choroidal volume (ChV) was greater in RVO eyes than that in fellow eye (FE). Moreover, ChV decreased after intravitreal bevacizumab treatment. Rayess et al. [38] showed how greater ChV tends to respond better to intravitreal injections, suggesting this parameter as a predictive biomarker to anti-VEGF therapy. In contrast to ChV, studying vessel density of choroidal layer in RVO patients showed no differences between baseline and after anti-VEGF treatment [35]. The masking effect of macular edema could also in this case be a limitation to data acquisition [13].

Few CRVO cases have been studied in terms of vessel density variation, usually analyzed in a mixed sample with BRVO and frequently rejected when the macular edema was severe [34,35]. In our experience, we run into the same limitations: poor quality images for inadequate patient fixation, unreliable OCTA data for impaired automated segmentation tool, and consequently difficult analytics interpretation. For CRVO involving the entire posterior pole retina, we decided to analyze other retinal sections than the macular region: papillary and peripapillary sectors.

Peripapillary retina has been studied in glaucoma disease because it is believed that this sector is primarily involved in glaucomatous damage, affecting both the retinal ganglion cells (RGCs) and the RNFL. OCTA technology allows structural and functional examination of RNFL thickness and peripapillary plexus in glaucoma, showing a lower peripapillary VD compared with healthy controls. Other authors found a decreased macular VD in glaucomatous eyes, suggesting wider retinal involvement [27,39]. However, at current time, there are no studies investigating papillary and peripapillary VD changings at OCTA in other pathologies, such as RVO. In this study we investigated for the first time microvascular papillary and peripapillary changes developing in CRVO before and after a loading phase of intravitreal anti-VEGF treatment. All our cases presented an history of arterial hypertension; high blood pressure represent a relevant risk factor in RVO which is considered as an end-organ damage in hypertensive disease. Structural OCT data demonstrated a reduction of CRT after intravitreal injections and no significant change in RNFL thickness. The fact that, in our cohort, mean RNFL thickness did not present significant difference after treatment shows how this area is less influenced by thickness variations compared to the macular region. After the 4^th^ month follow-up, patients had a significantly improved BCVA. Using OCTA technology, we found a significative inside disc and peripapillary VD increase after injections therapy.

A recent study showed that VD measurement by OCTA presents a good to moderate reproducibility in peripapillary region and a good reproducibility in papillary region, validating this area as a well-founded field of interest [40]. Manalastas et al. [41] described a similar good reproducibility of OCTA optic nerve head measurements using Optovue software. Considering possible fluctuation in VD measurement inter- and intra-subject, we tried to limit known determinants [42]. All data was obtained during a specific range of time on the day that was the same for every patient limiting the diurnal VD change. Our subjects did not presented glaucoma or raised IOP, nor myopia that are, according to the literature, factors that reduce the repeatability of the examination. The hypertensive disease in this case is a common characteristic limiting the subject-related factor. The attention focused on VD measurement is aimed to valorize our results which, however small and not determinant in clinical practice yet, show a trend in inside disc and peripapillary VD increase after anti-VEGF therapy.

Data of fellow eyes were analyzed and compared with the study eyes. Fluctuations of VD in the fellow eyes during the follow-up period were not statistically significant and VD was significantly higher in the unaffected eye at baseline, 1 month, and 4 months after treatment. It is most likely that there is a sudden reduction of inside disc and peripapillary VD after CRVO event, that slowly increases after anti-VEGF therapy. Moreover, VD parameter changes in the fellow eye were not statistically significant, showing a good exam reproducibility.

The limitations of the current study include motion artifacts from poor fixation, a limited number of enrolled patients and a short-time follow-up. Therefore, further studies using larger samples of patients, a longer follow-up time, and different devices are necessary to validate our results and to advance our understanding of microvascular changes associated with RVO.

However, this is the first time that a retinal region different from macula has been OCTA-analyzed in CRVO patients. These preliminary results could represent a first step in the study of a novel region of interest in patients with macular edema secondary to RVO.

## Figures and Tables

**Table 1:** Demographic characteristics and measurements at baseline, 1 and 4 months after ranibizumab intravitreal injection. T-test was performed to study continuous variables (*p*-value refers to parameter changes). Abbreviations: BCVA = best corrected visual acuity; logMAR = logarithm of the minimal angle of resolution; RNFL = retinal nerve fiber layer; RPC = radial peripapillary capillary; VD = vessel density. Bold numbers indicate a *p*-value of less than 0.05.

Parameters Observed	Baseline	1-Month Post-Operative	4-Months Post-Operative	*p*-Value (Baseline and 1-Month Post-Operative)	*p*-Value (Baseline - and 4-Months Post-Operative)
Age (years)	56.09 (16.47)				
Gender (male)	11 (61.11)				
**Study Eye**					
BCVA (logMAR)	0.70 (0.26)	0.34 (0.17)	0.25 (0.18)	<0.001	<0.001
RPC Vessel density (%)					
Whole image	44.64 (2.70)	45.34 (2.63)	45.75 (4.56)	0.09	0.08
Inside disc	45.16 (2.33)	45.71 (2.03)	47.08 (2.58)	0.23	<0.001
Peripapillary	43.84 (1.74)	44.78 (2.11)	45.58 (2.86)	0.04	0.01
RNFL thickness	182.06 (70.89)	172.11 (44.12)	151.83 (32.69)	0.3	0.05
Macular thickness	772.61 (199.54)	435.56 (105.42)	421.28 (111.66)	<0.001	<0.001
**Control Eye**					
RPC Vessel density (%)					
Whole image	50.16 (1.53)	50.04 (1.44)	50.09 (1.35)	0.41	0.74
Inside disc	50.33 (1.56)	50.38 (1.49)	50.44 (1.40)	0.61	0.41
Peripapillary	51.12 (1.85)	51.02 (1.61)	51.27 (2.82)	0.49	0.79

## References

[1] Ip M., Hendrick A. (2017). Retinal Vein Occlusion Review. Asia-Pac. J. Ophthalmol..

[2] Battaglia Parodi M., Bandello F. (2009). Branch Retinal Vein Occlusion: Classification and Treatment. Ophthalmologica.

[3] Aydin Kaderli A., Kaderli B., Gullulu S., Avci R. (2010). Impaired Aortic Stiffness and Pulse Wave Velocity in Patients with Branch Retinal Vein Occlusion. Graefe’s Arch. Clin. Exp. Ophthalmol..

[4] Gasparyan A.Y., Ayvazyan L., Mikhailidis D.P., Kitas G.D. (2011). Mean Platelet Volume: A Link between Thrombosis and Inflammation?. Curr. Pharm. Des..

[5] Bawankar P., Samant P., Lahane T., Parekh R., Lahane S. (2019). Mean Platelet Volume and Central Retinal Vein Occlusion in Hypertensive Patients. Can. J. Ophthalmol..

[6] Dodson P.M., Westwick J., Marks G., Kakkar V.V., Galton D.J. (1983). β-Thromboglobulin and Platelet Factor 4 Levels in Retinal Vein Occlusion. Br. J. Ophthalmol..

[7] Jaulim A., Ahmed B., Khanam T., Chatziralli I.P. (2013). Branch Retinal Vein Occlusion: Epidemiology, Pathogenesis, Risk Factors, Clinical Features, Diagnosis, and Complications. An Update of the Literature. Retina.

[8] Cugati S., Jie J.W., Rochtchina E., Mitchell P. (2006). Ten-Year Incidence of Retinal Vein Occlusion in an Older Population: The Blue Mountains Eye Study. Arch. Ophthalmol..

[9] Sakimoto S., Kamei M., Suzuki M., Yano S., Matsumura N., Sakaguchi H., Gomi F., Nishida K. (2012). Relationship between Grades of Macular Perfusion and Foveal Thickness in Branch Retinal Vein Occlusion. Clin. Ophthalmol..

[10] Suzuki N., Hirano Y., Yoshida M., Tomiyasu T., Uemura A., Yasukawa T., Ogura Y. (2016). Microvascular Abnormalities on Optical Coherence Tomography Angiography in Macular Edema Associated with Branch Retinal Vein Occlusion. Am. J. Ophthalmol..

[11] Noma H., Funatsu H., Mimura T., Harino S., Hori S. (2009). Vitreous Levels of Interleukin-6 and Vascular Endothelial Growth Factor in Macular Edema with Central Retinal Vein Occlusion. Ophthalmology.

[12] Tan A.C.S., Tan G.S., Denniston A.K., Keane P.A., Ang M., Milea D., Chakravarthy U., Cheung C.M.G. (2018). An Overview of the Clinical Applications of Optical Coherence Tomography Angiography. Eye.

[13] Mastropasqua R., Toto L., Di Antonio L., Borrelli E., Senatore A., Di Nicola M., Di Martino G., Ciancaglini M., Carpineto P. (2017). Optical Coherence Tomography Angiography Microvascular Findings in Macular Edema Due to Central and Branch Retinal Vein Occlusions. Sci. Rep..

[14] Holló G. (2017). Optical Coherence Tomography Angiography to Better Understand Glaucoma. J. Curr. Glaucoma Pract..

[15] Or C., Sabrosa A.S., Sorour O., Arya M., Waheed N. (2018). Use of OCTA, FA, and Ultra-Widefield Imaging in Quantifying Retinal Ischemia: A Review. Asia-Pac. J. Ophthalmol..

[16] Parodi M.B., Visintin F., Rupe P.D., Ravalico G. (1995). Foveal Avascular Zone in Macular Branch Retinal Vein Occlusion. Int. Ophthalmol..

[17] Noma H., Funatsu H., Sakata K., Harino S., Nagaoka T., Mimura T., Sone T., Hori S. (2009). Macular Microcirculation and Macular Oedema in Branch Retinal Vein Occlusion. Br. J. Ophthalmol..

[18] Kang J.W., Yoo R., Jo Y.H., Kim H.C. (2017). Correlation of Microvascular Structures on Optical Coherence Tomography Angiography with Visual Acuity in Retinal Vein Occlusion. Retina.

[19] Rispoli M., Savastano M.C., Lumbroso B. (2015). Capillary Network Anomalies in Branch Retinal Vein Occlusion on Optical Coherence Tomography Angiography. Retina.

[20] Suzuki N., Hirano Y., Tomiyasu T., Esaki Y., Uemura A., Yasukawa T., Yoshida M., Ogura Y. (2016). Retinal Hemodynamics Seen on Optical Coherence Tomography Angiography before and after Treatment of Retinal Vein Occlusion. Invest. Ophthalmol. Vis. Sci..

[21] Campochiaro P.A., Bhisitkul R.B., Shapiro H., Rubio R.G. (2013). Vascular Endothelial Growth Factor Promotes Progressive Retinal Nonperfusion in Patients with Retinal Vein Occlusion. Ophthalmology.

[22] Wang X., Jiang C., Kong X., Yu X., Sun X. (2017). Peripapillary Retinal Vessel Density in Eyes with Acute Primary Angle Closure: An Optical Coherence Tomography Angiography Study. Graefe’s Arch. Clin. Exp. Ophthalmol..

[23] Kim S.B., Lee E.J., Han J.C., Kee C. (2017). Comparison of Peripapillary Vessel Density between Preperimetric and Perimetric Glaucoma Evaluated by OCT-Angiography. PLoS ONE.

[24] Sellam A., Glacet-Bernard A., Coscas F., Miere A., Coscas G., Souied E.H. (2017). Qualitative and Quantitative Follow-up Using Optical Coherence Tomography Angiography of Retinal Vein Occlusion Treated with Anti-Vegf. Retina.

[25] Liu L., Wang Y., Liu H.X., Gao J. (2019). Peripapillary Region Perfusion and Retinal Nerve Fiber Layer Thickness Abnormalities in Diabetic Retinopathy Assessed by OCT Angiography. Transl. Vis. Sci. Technol..

[26] Burgoyne C.F. (2011). A Biomechanical Paradigm for Axonal Insult within the Optic Nerve Head in Aging and Glaucoma. Exp. Eye Res..

[27] Lommatzsch C., Rothaus K., Koch J.M., Heinz C., Grisanti S. (2019). Retinal Perfusion 6 Months after Trabeculectomy as Measured by Optical Coherence Tomography Angiography. Int. Ophthalmol..

[28] Coscas F., Glacet-Bernard A., Miere A., Caillaux V., Uzzan J., Lupidi M., Coscas G., Souied E.H. (2016). Optical Coherence Tomography Angiography in Retinal Vein Occlusion: Evaluation of Superficial and Deep Capillary Plexa. Am. J. Ophthalmol..

[29] Seknazi D., Coscas F., Sellam A., Rouimi F., Coscas G., Souied E.H., Glacet-Bernard A. (2018). Optical Coherence Tomography Angiography in Retinal Vein Occlusion: Correlations between Macular Vascular Density, Visual Acuity, and Peripheral Nonperfusion Area on Fluorescein Angiography. Retina.

[30] Mastropasqua R., Di Antonio L., Di Staso S., Agnifili L., Di Gregorio A., Ciancaglini M., Mastropasqua L. (2015). Optical Coherence Tomography Angiography in Retinal Vascular Diseases and Choroidal Neovascularization. J. Ophthalmol..

[31] Adhi M., Bonini Filho M.A., Louzada R.N., Kuehlewein L., De Carlo T.E., Baumal C.R., Witkin A.J., Sadda S.R., Sarraf D., Reichel E. (2016). Retinal Capillary Network and Foveal Avascular Zone in Eyes with Vein Occlusion and Fellow Eyes Analyzed with Optical Coherence Tomography Angiography. Invest. Ophthalmol. Vis Sci..

[32] De Carlo T.E., Chin A.T., Joseph T., Baumal C.R., Witkin A.J., Duker J.S., Waheed N.K. (2016). Distinguishing Diabetic Macular Edema from Capillary Nonperfusion Using Optical Coherence Tomography Angiography. Ophthalmic Surg. Lasers Imaging Retina..

[33] Mané V., Dupas B., Gaudric A., Bonnin S., Pedinielli A., Bousquet E., Erginay A., Tadayoni R., Couturier A. (2016). Correlation between Cystoid Spaces in Chronic Diabetic Macular Edema and Capillary Nonperfusion Detected by Optical Coherence Tomography Angiography. In Retina.

[34] Winegarner A., Wakabayashi T., Fukushima Y., Sato T., Hara-Ueno C., Busch C., Nishiyama I., Shiraki N., Sayanagi K., Nishida K. (2018). Changes in Retinal Microvasculature and Visual Acuity after Antivascular Endothelial Growth Factor Therapy in Retinal Vein Occlusion. Invest. Ophthalmol. Vis Sci..

[35] Costanzo E., Parravano M., Gilardi M., Cavalleri M., Sacconi R., Aragona E., Varano M., Bandello F., Querques G. (2019). Microvascular Retinal and Choroidal Changes in Retinal Vein Occlusion Analyzed by Two Different Optical Coherence Tomography Angiography Devices. Ophthalmologica.

[36] Ghasemi Falavarjani K., Iafe N.A., Hubschman J.P., Tsui I., Sadda S.R., Sarraf D. (2017). Optical Coherence Tomography Angiography Analysis of the Foveal Avascular Zone and Macular Vessel Density after Anti-VEGF Therapy in Eyes with Diabetic Macular Edema and Retinal Vein Occlusion. Invest. Ophthalmol. Vis Sci..

[37] Samara W.A., Shahlaee A., Sridhar J., Khan M.A., Ho A.C., Hsu J. (2016). Quantitative Optical Coherence Tomography Angiography Features and Visual Function in Eyes with Branch Retinal Vein Occlusion. Am. J. Ophthalmol..

[38] Rayess N., Rahimy E., Ying G.S., Pefkianaki M., Franklin J., Regillo C.D., Ho A.C., Hsu J. (2016). Baseline Choroidal Thickness as a Predictor for Treatment Outcomes in Central Retinal Vein Occlusion. Am. J. Ophthalmol..

[39] Baek S.U., Kim Y.K., Ha A., Kim Y.W., Lee J., Kim J.S., Jeoung J.W., Park K.H. (2019). Diurnal Change of Retinal Vessel Density and Mean Ocular Perfusion Pressure in Patients with Open-Angle Glaucoma. PLoS ONE.

[40] Fernández-Vigo J.I., Kudsieh B., Macarro-Merino A., Arriola-Villalobos P., Martínez-de-la-Casa J.M., Feijóo J.G., Fernández-Vigo J.Á. (2019). Reproducibility of Macular and Optic Nerve Head Vessel Density Measurements by Swept-Source Optical Coherence Tomography Angiography. Eur. J. Ophthalmol..

[41] Manalastas P.I.C., Zangwill L.M., Saunders L.J., Mansouri K., Belghith A., Suh M.H., Yarmohammadi A., Penteado R.C., Akagi T., Shoji T. (2017). Reproducibility of optical coherence tomography angiography macular and optic nerve head vascular density in glaucoma and healthy eyes. J. Glaucoma.

[42] Moghimi S., Hou H., Rao H.L., Weinreb R.N. (2019). Optical Coherence Tomography Angiography and Glaucoma: A Brief Review. Asia-Pac. J. Ophthalmol..

